# Transcriptional regulation of the p73 gene by Nrf-2 and promoter CpG methylation in human breast cancer

**DOI:** 10.18632/oncotarget.2230

**Published:** 2014-07-17

**Authors:** Jing Lai, Weiwei Nie, Wenwen Zhang, Yucai Wang, Ruilian Xie, Yanru Wang, Jun Gu, Jing Xu, Wei Song, Fang Yang, Guichun Huang, Peng Cao, Xiaoxiang Guan

**Affiliations:** ^1^ Department of Medical Oncology, Jinling Hospital, School of Medicine, Southern Medical University, Guangzhou, China; ^2^ Department of Medical Oncology, Jinling Hospital, Medical School of Nanjing University, Nanjing, China; ^3^ Department of Medicine, Rutgers New Jersey Medical School, Newark, NJ, USA; ^4^ Department of General Surgery, Jinling Hospital, Medical School of Nanjing University, Nanjing, China; ^5^ Laboratory of Cellular and Molecular Biology, Jiangsu Province Institute of Chinese Medicine, Nanjing, China

**Keywords:** breast cancer, TAp73, ΔNp73, Nrf-2, methylation, transcription

## Abstract

To understand the transcriptional regulation of p73 by promoter methylation and Nrf-2 in breast carcinogenesis, ChIP assay indicated that Nrf-2 can bind to both promoters and can activate the transcription of TAp73 and ΔΝp73 in MCF-7 cell line, knockdown of Nrf-2 gene resulted in an abrogation of TAp73 and ΔΝp73 expression in the cells transfected with sh-Nrf-2 as well as Nrf-2 knock out mouse model. However, we found Nrf-2 induced ΔΝp73 expression was abolished with 5-aza-dC treatment, thus lead to a down-regulated ΔΝp73 and an up-regulated TAp73 expression in breast cancer cells lines. Consistent with this model, we detected decreased TAp73 and increased ΔNp73 expression in breast cancer tissue, along with increased TAp73 but decreased ΔNp73 expression in corresponding surrounding noncancerous tissues (NCTs) in a breast cancer tissue assay. A significant inverse correlation was found between TAp73 and ΔNp73 expression in the above tissue-array (*P* = 0.047) and validated in another set consisting of 128 breast cancer tumor tissue (*P* = 0.034). Taken together, our findings suggest that Nrf-2 and promoter methylation cooperatively govern the transcriptional regulation of p73, and unbalanced expression of TAp73 and ΔNp73 expression plays a critical role in breast cancer development.

## INTRODUCTION

The p73 gene has two distinct promoters coding for two major isoforms, full-length TAp73 and the amino-terminally truncated ΔNp73, respectively [1-3]. The TAp73 isoform encodes proteins that are often activated following DNA damage and promote cell death [4, 5], whereas the ΔNp73 isoform lacking the transactivation domain acts as an oncogene by triggering intracellular signaling cascades leading to cell transformation and tumorigenicity [6, 7]. Functional report shows that mice with a selective deficiency of TAp73 develop spontaneous tumors, particularly lung adenocarcinomas, and are more sensitive to chemical carcinogenesis [8]. Meanwhile, several studies found that overexpression of ΔNp73 in many human cancers has been shown to inhibit apoptosis [9-11], and increased levels of ΔNp73 in primary tumors have been shown to correlate with poor prognosis [10, 12-14]. Therefore, accumulating evidences support the fact that the p73 gene plays critically important role in the development and progression of malignant cancer. However, little is known regarding the transcriptional and functional regulation of the p73 gene in breast cancer.

Reports have showed that methylation of p73 gene is observed in hematological malignancies [15, 16] and some solid tumors such as lung cancer [17], gastric carcinoma [18] and cervical cancer [19], since alterations of the pattern of DNA methylation can lead to silencing of tumor suppressor genes [20, 21]. And the re-expression of p73 occurred as a consequence of promoter demethylation by DNA Methyltransferase (DNMTs) inhibitor 5-aza-2′-deoxycytidine (5-aza-dC), which reverted basic methylation of CpGs to an unmethylated status [22], widely used in demethylation studies and clinical practice to reverse DNA methylation [23]. It also has been reported that the extrinsic P1 promoter and intrinsic P2 promoter are differentially affected by methylation [17, 24, 25]. However, the methylation states of the two promoters and the relative contribution of gene reactivation with 5-aza-dC in breast cancer are not entirely revealed.

It had been reported that the P1 promoter contains functional E2F1-binding sites [26], through which the E2F1 transcription factor can induce TAp73 overexpression and led to apoptosis [27]. However, the study also reported that the P1 promoter is not completely inactivated after the treatment of site-directed mutagenesis to its functional E2F1 sites. And we asked whether additional transcription factor(s) play a signiﬁcant role in the regulation of p73 gene. In present work, we focused on the identification of novel transcriptional factors that can regulate the expression of p73 gene.

Using bioinformatic analysis to predict transcription factors binding sites in p73 promoters, we found that both P1 and P2 promoter have the putative binding sites for nuclear factor erythroid 2-related factor 2 (Nrf-2) and contain CpG methylation islands. Importantly, we demonstrated that Nrf-2 can regulate the transcription of the p73 gene by specifically bind to the P1 and P2 promoter and 5-aza-dC treatment can led to an increased binding with P1 and decreased binding with P2 promoter. To the best of our knowledge, this is the first report revealing that Nrf-2 takes opposite effect in the demethylation-induced p73 isoforms transcriptional regulation in human breast cancer. This finding would provide insights into the potential target for the future therapy of breast cancer.

## RESULTS

### 5-aza-dC induces cell proliferation inhibition along with cell cycle arrest and apoptosis in breast cancer cell lines

**Figure 1:**
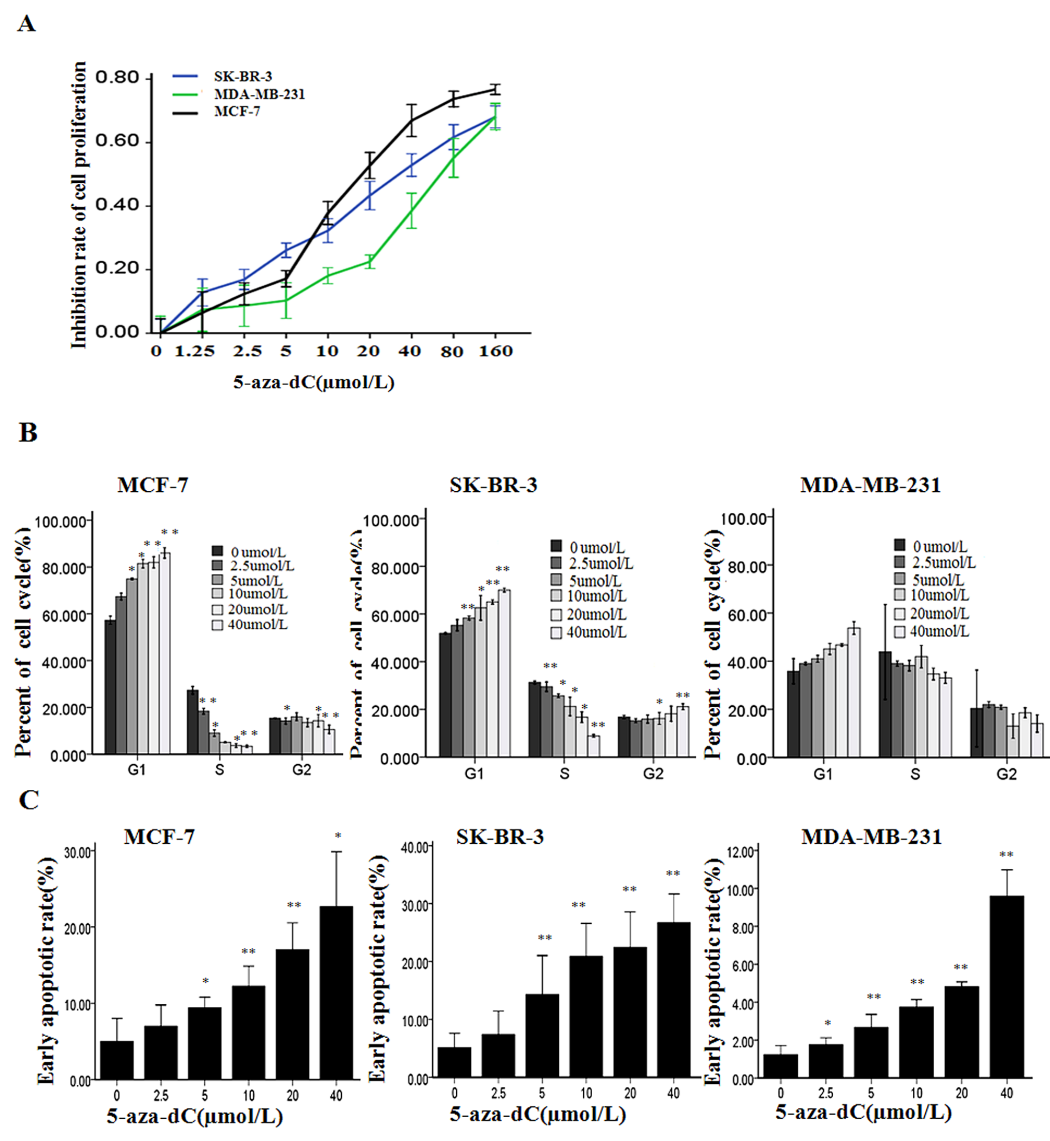
5-aza-dC induces cell proliferation inhibition along with cycle arrest and apoptosis (A) Cell viability were measured with MTT after MCF-7, SK-BR-3 and MDA-MB-231 were treated with 5-aza-dC at various concentrations for 48h. Results are presented as the mean ± SD of triplicate observations, P<0.05 controlled with untreated group in the three cell lines. (B) Cell cycle distribution was determined by flow cytometry analysis using PI staining after treatment with 5-aza-dC for 48h at the indicated concentrations. (C) Cell apoptosis was detected by flow cytometry analysis after the cells were treated by the same treatment as B. Results are presented as the mean ± SD of triplicate observations. *P < 0.05 or **P < 0.001.

To investigate the effect of 5-aza-dC in breast cancer, we analyzed the induction of cell proliferation inhibition in three breast cancer cell lines including MCF-7, SK-BR-3 and MDA-MB-231 treated with increasing concentrations of 5-aza-dC (0–160μmol/L) for 48 h. As shown in Figure 1A, dose-dependent inhibitions of cell proliferation were observed in the three breast cancer cell lines, and the cell viability was decreased by about 50% when the MCF-7 cells was treated with the 5-aza-dC at 20μmol/L for 48 h (*P*< 0.05). Hence, we selected the MCF-7 cells treated with 0-20μmol/L 5-aza-dC for 48 h for the further studies.

Report had showed that the growth inhibition of 5-aza-dC was attributed to its ability to arrest cells at the G_1_ and G_2_-M phases of cell cycle [28]. In present study, to explore the potential role of 5-aza-dC in the cell cycle in breast cancer cells, we used flow cytometry to determine the percentage of cells in G_1_, S, and G_2_ compartments of cell cycle from each group treated with various concentrations of 5-aza-dC for 48 h. As shown in Figure 1B, the cell cycle distribution analysis of the MCF-7 and SK-BR-3 cells showed a dose-related increase in G_1_ phase and a dose-related decrease in S phase of the 5-aza-dC treated cells, respectively, in comparison with the untreated control (*P*<0.05). Though, the same tendency was captured in the MDA-MB-231 cells, however, these changes were no statistically significant.

**Figure 2:**
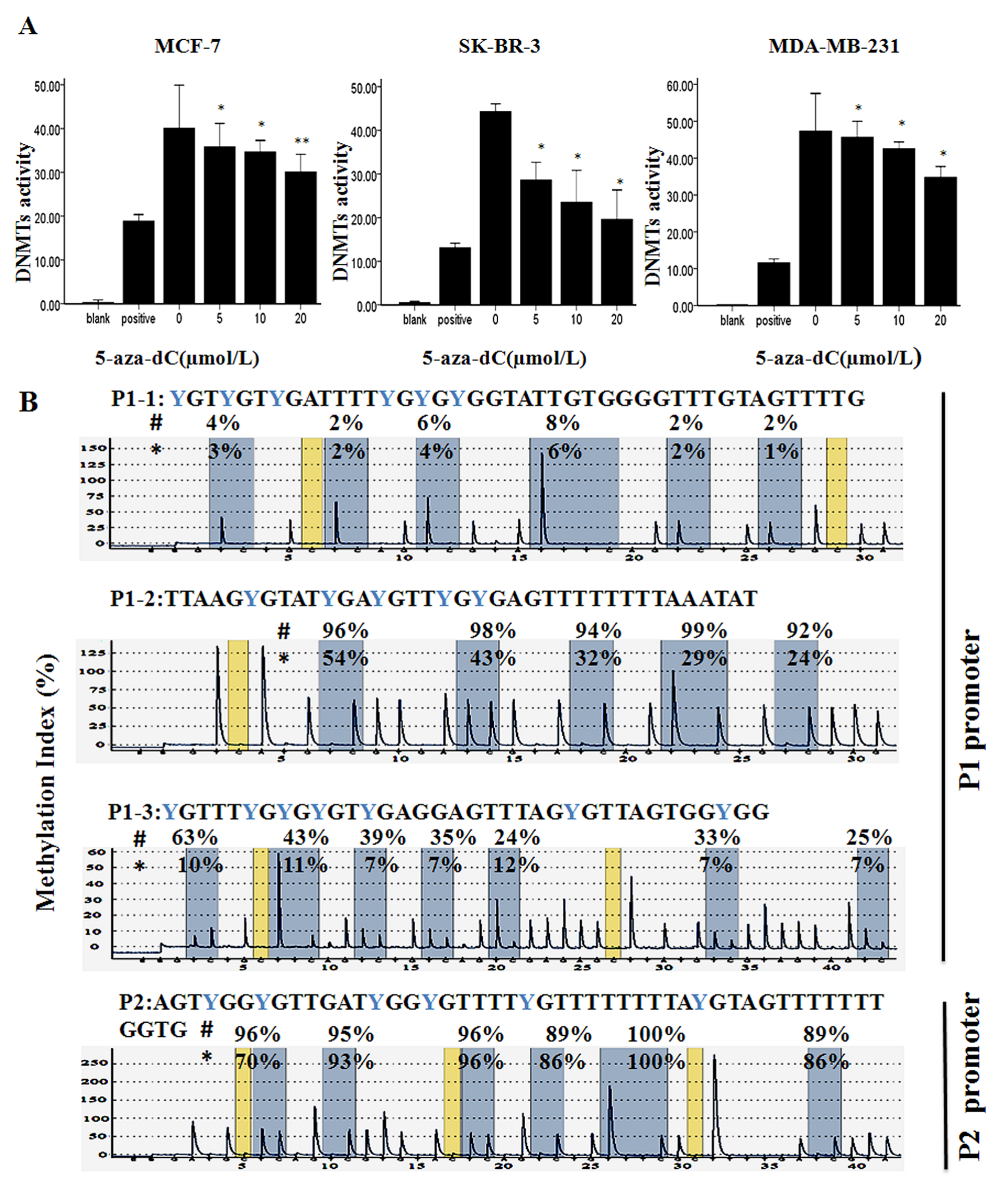
Effect of 5-aza-dC on DNMTs activity and Methylation status of P1 and P2 promoters (A) Total DNMT activity was evaluated in MCF-7, SK-BR-3 and MDA-MB-231 after treatment with 0-20μmol/L 5-aza-dC for 48h. Results are the mean remaining DNMT activity ± relative error of three independent experiments. *P < 0.05 or **P < 0.001 vs. untreated controls. (B) The methylation state of the P1 promoter and P2 promoter were detected by Pyrosequencing in MCF-7 after treatment with DMSO (#) or 20μmol/L 5-aza-dC (*) for 48h. Gray columns depict regions of CpG sites, and the percentage methylation at each CpG site is shown on the top. The percentage of methylation is calculated as the C/(C + T) peak ratio per CpG.

To further confirm the nature of the cell death, we used the Annexin V flow cytometry assay to detect the cell apoptosis after the cells were exposed to various concentrations of 5-aza-dC for 48 h (Figure 1C). It has been shown that exposure to 5-aza-dC caused cell apoptosis in a dose-dependent fashion in the three breast cancer cells compared with control, respectively (P<0.05). As shown in Figure 2A, 5-aza-dC treatment inhibited DNMTs activity in breast cancer cells. And pyrosequencing assay successfully detected enrich CPG islands in P1 and P2, and indicated that both P1 and P2 promoters were methylated in breast cancer cell lines, which could be reversed by 5-aza-dC treatment (Figure 2B). In addition, bisulfite sequencing analysis (BSP) was conducted on bisulfite-modified DNA from P2 in MCF-7 cells cultured with 20 μmol/L 5-aza-dC or DMSO (Supplementary Figure S1).

### 5-aza-dC induces the expression of TAp73 and inhibits the expression of ΔNp73 in MCF-7 cell line

**Figure 3:**
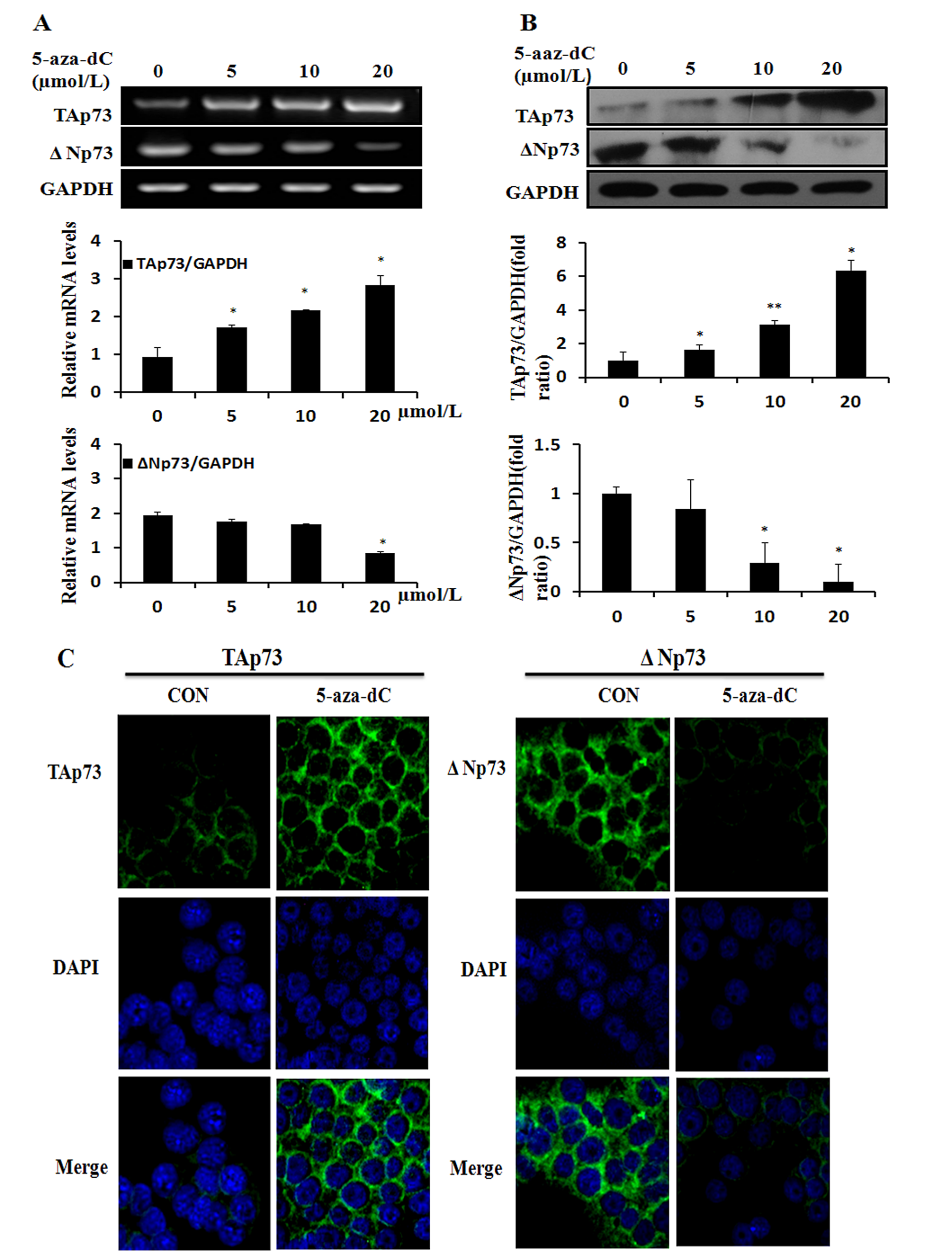
5-aza-dC induces the expression of TAp73 and inhibits the expression of ΔNp73 (A) TAp73 mRNA and ΔNp73 mRNA expression were determined by RT-PCR in MCF-7 after treatment with 0-20μmol/L 5-aza-dC for 48h, and fold changes in intensity normalized by GAPDH were shown by densitometric analysis. Results represented the average of three independent experiments. *P<0.05, as compared with the untreated cells. (B) and the TAp73 and ΔNp73 protein were determined by Western blotting after the same 5-aza-dC treatment, and GAPDH loading control. *P<0.05 or **P<0.001. (C) and the localization and expression of TAp73 and ΔNp73 were determined by fluorescent microscopy after treatment with 20μmol/L 5-aza-dC for 48h, and DMSO treatment loading control. Nuclei were stained with 4,6-diamidino-2-phenylindole (DAPI).

To investigate the effect of 5-aza-dC on the expression of p73 gene in MCF-7 cell line, we first exposed MCF-7 cells to various concentrations (5, 10, 20μM) of 5-aza-dC for 48h, and assessed the TAp73 and ΔNp73 mRNA levels by RT-PCR and protein levels by Western blot analysis (Figure 3). As shown in Figure 3A, 5-aza-dC treatment led to an increased TAp73 and a decreased ΔNp73 expression in MCF-7 cells, which was opposite to the change of DNA methylation levels of P2 promoter in MCF-7 when treated with 5-aza-dC. Meanwhile, 5-aza-dC treatment in MCF-7 cells caused an up-regulation of TAp73 and a down-regulation of ΔNp73, and the change of protein levels was in keeping with the expression of mRNA (Figure 3B). Taken together, our data showed that TAp73 expression could be induced and ΔNp73 expression could be inhibited by 5-aza-dC both in transcriptional and translational levels.

Furthermore, immunofluorescent staining was used to verify the expression and examine the subcellular localization of protein (Figure 3C), and we detected TAp73 and ΔNp73 in somatoplasm, TAp73 expression (green; left panels) was significantly enhanced in the MCF-7 cells after treatment with 20umol/L 5-aza-dC for 48h when contrasted with the untreated cells, while the expression of ΔNp73 (green; right panels) was weakened. This result was consistent with the protein expression which had been confirmed by Western blotting.

### TAp73 and ΔNp73 are regulated by Nrf-2 through a regulatory region in the different promoters of p73 gene

**Figure 4:**
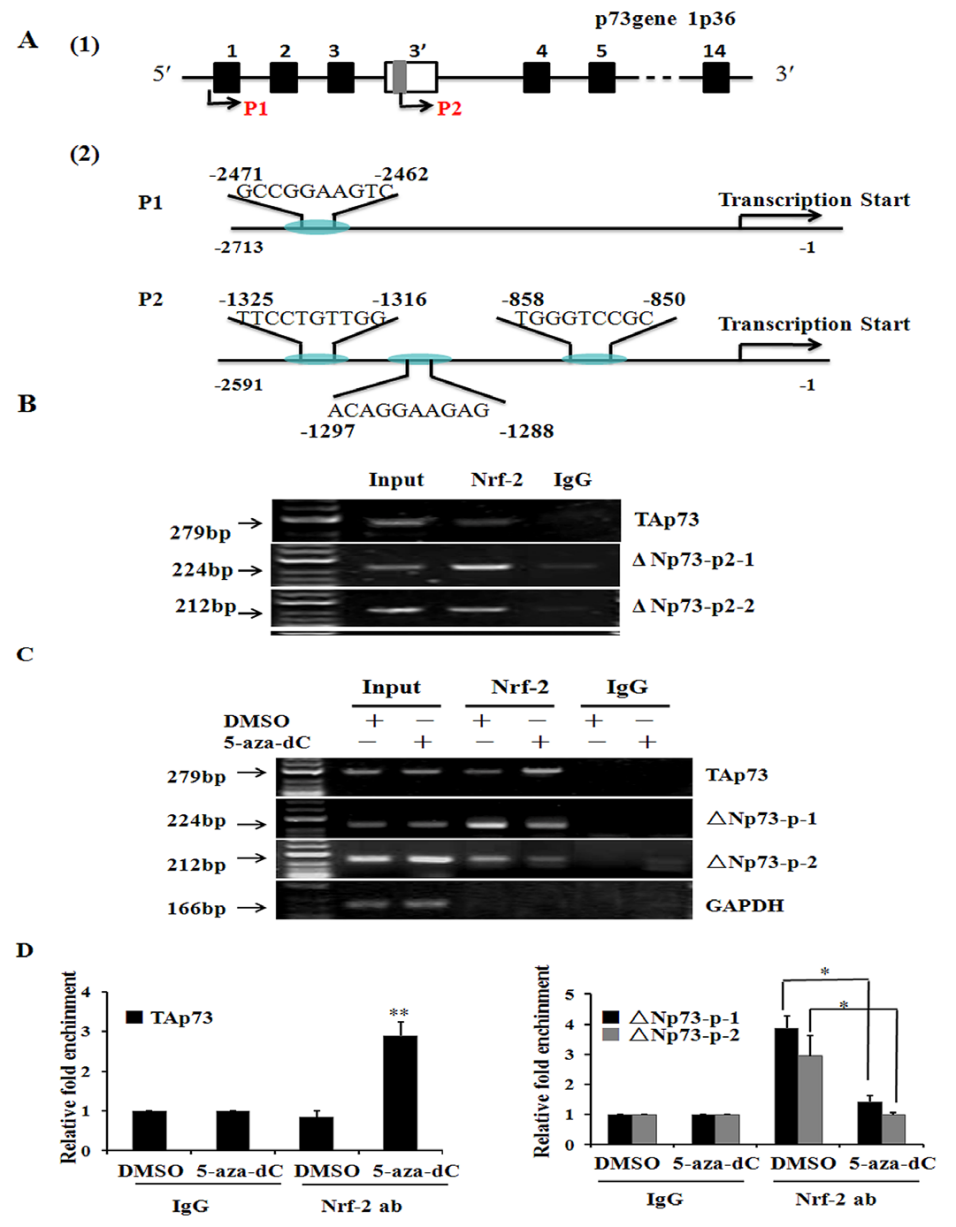
TAp73 and ΔNp73 are regulated by Nrf-2 through a regulatory region in p73 different promoters (A) Schematic model of Nrf-2 binding sites in P1 promoter and P2 promoter of p73 gene by bioinformatic analysis. The sites and sequences of three binding sites were indicated in model scheme. (B) ChIP assay showed that Nrf-2 can specifically bind to the TAp73 and ΔNp73 in MCF-7 cells. (C) ChIP assay showed that Nrf-2 can specifically bind to the TAp73 and ΔNp73 in MCF-7 cells after treatment with DMSO and 5-aza-dC for 24h. The relative binding of Nrf-2 to P2 promoter was quantified from the band intensities of three independent experiments and plotted. (D) and quantitative real time PCR showed 5-aza-dC treatment increase the binding of Nrf-2 to P1 promoter and inhibited the binding of Nrf-2 to P2 promoter. The mixture was run on a 7500 Real-Time PCR System (Applied Biosystems) using relative quantization according to the manufacturer's protocols. The amounts of immunoprecipitated DNA were normalized to the inputs and plotted. *P < 0.05 or **P<0.001.

Bioinformatic analysis was used to predict transcription factors binding sites in p73 promoters, indicating that both P1 and P2 promoter have the putative binding sites for Nrf-2 and contain CpG methylation islands. As shown in Figure 4A, one binding sites in P1 promoter and three binding sites in P2 promoter (named A, B and C, respectively) were predicted. Then, we designed one and two sites by Applied Biosystems to detect binding between Nrf-2 and P1 or P2 promoter, respectively. The ΔNp73-p-1 was the first site including sequence A and sequence B; ΔNp73-p-2 was the second site including sequence C. To determine whether Nrf-2 bind to the promoters of p73 gene and regulated the expression of TAp73 and ΔNp73, we performed Chromatin immunoprecipitation (ChIP) assays and Q-PCR using custom-made probes and primers to measure the relative bindings of Nrf-2 to the region of P1 and P2 promoter, respectively. And the results of ChIP assays clearly demonstrated that Nrf-2 could specifically bind to the TAp73 and ΔNp73 at all of the three binding sites in MCF-7 cells (Figure 4B). However, the Nrf-2 binding to TAp73 was enhanced, while the Nrf-2 binding to ΔNp73 was weakened in response to treatment with 20μmol/L 5-aza-dC for 24h; and it was absent when chromatin was immunoprecipitated with control IgG (Figure 4C). Furthermore, the relative bindings of Nrf-2 to TAp73 and ΔNp73 were measured and plotted, which also showed that the treatment of 5-aza-dC can enhance the Nrf-2 binding to TAp73 and inhibit the Nrf-2 binding to ΔNp73 (Figure 4D). Collectively, our data confirmed that TAp73 and ΔNp73 are regulated by Nrf-2 through a regulatory region in the different promoters of p73 gene, and treatment with 5-aza-dC can enhance Nrf-2 to bind to the P1 promoter and inhibit Nrf-2 to bind to the P2 promoter resulting in the regulation of p73 expression.

### The up-regulation of TAp73 and down-regulation of ΔNp73 induced by 5-aza-dC are correlated with Nrf-2 expression *in vitro* and *in vivo*

**Figure 5:**
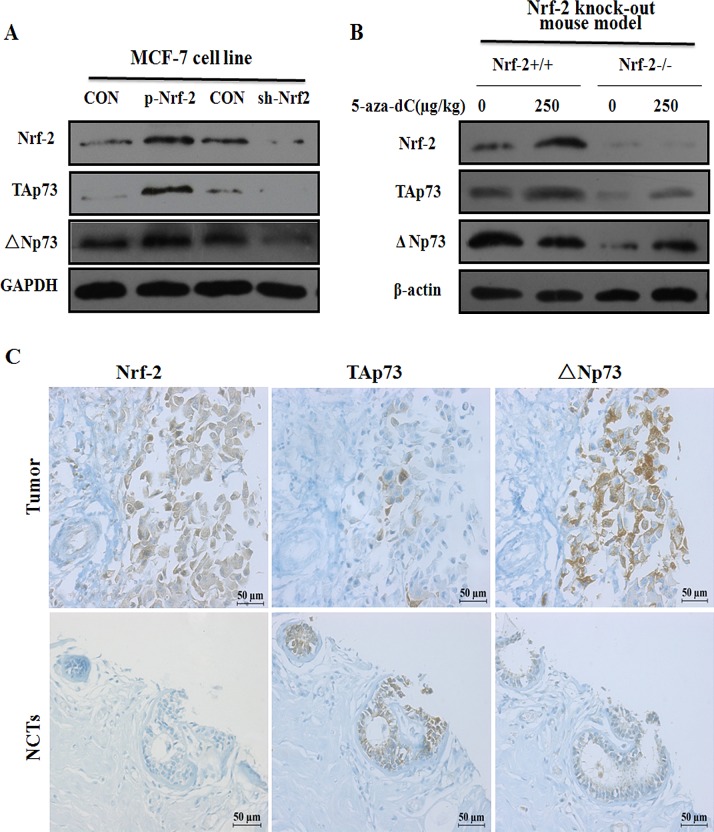
TAp73 and ΔNp73 expression are correlated with 5-aza-dC induced Nrf-2 expression *in vitro* and *in vivo* (A) The protein expression of TAp73 and ΔNp73 were detected by Western blotting after transfection with p-Nrf-2 or sh-Nrf-2 for 24h in MCF-7. The relative protein levels for them were represented by column graph when normalized to GAPDH expression. (B) The TAp73 and ΔNp73 expression from mammary gland in Nrf-2 knock out mice model were detected by Western blotting after injected through tail vein with 250ug/kg 5-aza-dC for 24h, and normalized to β-actin expression. Results represented the average of three independent experiments. *P<0.05, as compared with the untreated mice. (C) Decreased expression of TAp73 and increased expression of ΔNp73 and Nrf-2 in breast cancer compared to normal and adjacent tissues was showed by immunohistochemical analysis of breast cancer tissues and corresponding noncancerous tissues (NCTs) tissue-array. Immunostained sections (brown) with indicated antibody above were counterstained with hematoxylin stain (blue). The high magnification (200×) regions shown above were indicated in the NCTs.

Since ChIP assays revealed that Nrf-2 could bind to P1 and P2 promoter in MCF-7 cells, we asked whether the expression of TAp73 and ΔNp73 was controlled by Nrf-2. To this end, after transiently transfected MCF-7 cells with p-Nrf-2 and sh-Nrf-2, we found that Nrf-2 transfection induced TAp73 and ΔΝp73 expression in MCF-7 cell line, knockdown of Nrf-2 gene resulted in an abrogation of TAp73 and ΔΝp73 expression in the cells transfected with sh-Nrf-2 suggesting a positive regulation of P1 and P2 by Nrf-2 transcriptional factor (Figure 5A).

To further determine whether the up-regulation of TAp73 and down-regulation of ΔNp73 induced by 5-aza-dC were correlated with Nrf-2 expression *in vivo*, using an Nrf-2 gene knock-out mice model, we detected the expression of TAp73 and ΔNp73 by western blotting analysis. As shown in Figure 5B, the TAp73 and ΔNp73 protein levels were significantly decreased in mammary gland of Nrf-2 −/− mice model when compared with Nrf-2 +/+ mice model. However, Nrf-2 induced ΔΝp73 expression was abolished with 5-aza-dC treatment. Collectively, these results suggested that Nrf-2 contributed to the expression of p73 by binding to P1 and P2 promoter and maybe act as a role to regulate p73 transcriptional activation in 5-aza-dC induced mammary gland. Taken together, our data showed that the up-regulation of TAp73 and down-regulation of ΔNp73 induced by 5-aza-dC are correlated with Nrf-2 expression *in vitro* and *in vivo*.

### An inverse significant correlation of the expression between TAp73 and ΔNp73

Since Nrf-2 could regulate the expression of p73 by binding to P1 and P2 promoter, we performed immunohistochemistry staining (IHC staining) using a tissue microarray to further explore the particular relationship of TAp73, ΔNp73 and Nrf-2 expression in the malignant tumor and corresponding surrounding noncancerous tissues (NCTs) samples from 55 patients with breast cancer (Supplementary Table S1). Representative IHC staining of TAp73, ΔNp73 and Nrf-2 in breast tissues was shown in Figure 5C and statistical analysis was shown in Table 1. We found that a decreased TAp73 and an increased ΔNp73 expression in breast cancer tissue than the NCTs (*P*=0.001 or <0.001). In the meantime, the expression of Nrf-2 expression in breast cancer tissue was also higher than in the NCTs when tested by Two-Related-samples test (*P* <0.001).

**Table 1:** TAp73, ΔNp73 and Nrf-2 expression in breast cancer and NAT microarray

	Tumor (n=55)		NAT (n=55)		*P*^a^
	No.	%	No.	%	
TAp73					0.001
−	41	74.5	25	45.5	
+	13	23.6	24	43.6	
++	0	0	6	10.9	
+++	1	1.8	0	9	
ΔNp73					<0.001
−	14	25.5	33	60	
+	5	9.1	21	38.2	
++	25	45.5	1	1.8	
+++	11	20	0	0	
Nrf-2					<0.001
−	16	29.1	34	61.8	
+	28	50.9	18	32.7	
++	8	14.5	3	5.5	
+++	3	5.5	0	0	

Tumor: breast cancer; NAT: normal and adjacent tissues.

a TAp73, ΔNp73 or Nrf-2 expression was tested by Two-Related-samples test between breast cancer and NAT.

To elucidate the down-regulation of TAp73 and up-regulation of ΔNp73 in tumor tissues, we also analyzed the correlation of the expression between TAp73 and ΔNp73 at the tumor microarray or at tumor tissues from 128 breast cancer patients to validate it, respectively (Table 2). Significantly, the inverse significant correlation was found between TAp73 and ΔNp73 expression in this breast cancer tissue-array (r=-0.269, p=0.047) and validated in another 128 breast cancer patient tumor tissue (r=-0.188, p=0.034). This possibly means that in tumor tissue, the down-regulation of TAp73 expression could be caused by subsequent up-regulation of ΔNp73 expression. Together with that TAp73 expression could be induced and ΔNp73 expression could be inhibited by 5-aza-dC both in transcriptional and translational levels (Figure 2), we proposed that TAp73 isoforms can regulate the transcription of ΔNp73 isoforms, which, in turn, act as dominant negative regulators of TAp73, thus giving a dominant negative feedback loop [29].

**Table 2:** Correlative analysis the expression between TAp73 and ΔNp73 at tumor microarray or at tumor tissues

	Testing Set		Validation Set	
	Tumor microarray (n=55)		Tumor tissues (n=128)	
	TAp73 (Positive)	TAp73 (Negative)	TAp73 (Positive)	TAp73 (Negative)
ΔNp73(Positive)	6	38	16	70
ΔNp73(Negative)	8	3	15	27
r	-0.269		-0.188	
*P*	0.047		0.034	

## DISCUSSION

DNA methylation, a dynamic and reversible mode of epigenetic regulation, can modify functionality of numerous genes by regulating DNMTs’ activities. 5-aza-dC (an inhibitor of DNMTs) can reactivate aberrantly hypermethylated genes by preventing maintenance of the methylation state, thereby playing antineoplastic roles by inducing apoptosis, cell cycle arrest, and differentiation [28, 30]. To investigate the effect of 5-aza-dC in breast cancer, we analyzed the induction of cell proliferation inhibition, determined the percentage of cells in G_1_, S, and G_2_ compartments of cell cycle and detected the cell apoptosis after treatment of 5-aza-dC in three breast cancer cell lines. As shown in Figure 1, we found that 5-aza-dC was capable of inhibiting the proliferation of breast cancer cells in inducing cell cycle arrest and apoptosis which was consistent with recent studies [31, 32]. Moreover, we observed that the DNMT activity in the three cell lines was detected a significant dose-dependent decrease after treatment with 5-aza-dC (Figure 2A), also we detected that both P1 and P2 promoters were methylated, which could be reversed by 5-aza-dC treatment (Figure 2B). Besides, our data showed that TAp73 expression could be induced and ΔNp73 expression could be inhibited by 5-aza-dC both in transcriptional and translational levels in MCF-7 cell line (Figure 3).

Previous reports have demonstrated that P1 promoter contains functional E2F1-binding sites [26], through which the E2F1 transcription factor can induce TAp73 overexpression and led to apoptosis [27]. Recently, it has been also shown that TAp73 isoforms are overexpressed in response to overexpression of Sp1 transcription factor, which directly activates P1 promoter in lung cancer [33]. Moreover, in present study, bioinformatic and functional analysis confirmed that both P1 and P2 promoter have the putative binding sites for Nrf-2 and contain CpG methylation islands. For the first time, our data demonstrated that Nrf-2 can specifically bind to the P1 and P2 promoter and 5-aza-dC treatment led to an increased binding with P1 and decreased binding with P2 promoter. Furthermore, Nrf-2 transfection can induce TAp73 and ΔΝp73 expression in MCF-7 cell line, while knockdown of Nrf-2 gene resulted in an abrogation of TAp73 and ΔΝp73 expression in the cells transfected with sh-Nrf-2 as well as Nrf-2 knock out mouse model (Figure 5A and B). Collectively, these results strongly suggested a positive regulation of P1 and P2 by Nrf-2 transcriptional factor. However, Nrf-2 induced ΔΝp73 expression was abolished with 5-aza-dC treatment, thus lead to an up-regulated TAp73 and a down-regulated ΔΝp73 expression in breast cancer cells lines. Additionally, using a tissue microarray in 55 breast cancer patients’ sample, we found that a decreased TAp73 and an increased ΔNp73 expression were observed in breast cancer tissue. In the meantime, the expression of Nrf-2 expression in breast cancer tissue was also higher than in the NCTs (Table 1). Coincidentally, it have been reported that the down-regulation of Nrf-2 appears to lead to defect in the cellular defense system against oxidative stress, which potentially resulted in increased reactive oxygen species and DNA damage [34, 35]. Altogether, given the established role of TAp73 and ΔΝp73 in breast cancer, the results provided an important mechanism for Nrf-2 to further influence oncogenesis and progression of breast carcinoma cells.

In agreement with previous studies [29, 36, 37], an inverse significant correlation was found between TAp73 and ΔNp73 expression in this tissue microarray and validated in breast cancer patient tumor tissue (Table 2), which can demonstrate that TAp73 and ΔΝp73 can regulate each other, keeping the trigger of cell death under tight control. Consequently, the ultimate effect of p73 isoforms in cancer progression is intrinsically attributed to the balance between TAp73 and ΔNp73, rather than the overexpression of a speciﬁc p73 isoform or a speciﬁc class of p73 isoforms [5, 38]. Therefore, the selective promoter activation could lead to the activation of either pro-apoptotic or anti-apoptotic isoform(s) of p73 gene, thereby shifting the TA/ΔN equilibrium towards an oncogenic or a tumor suppressor direction.

**Figure 6:**
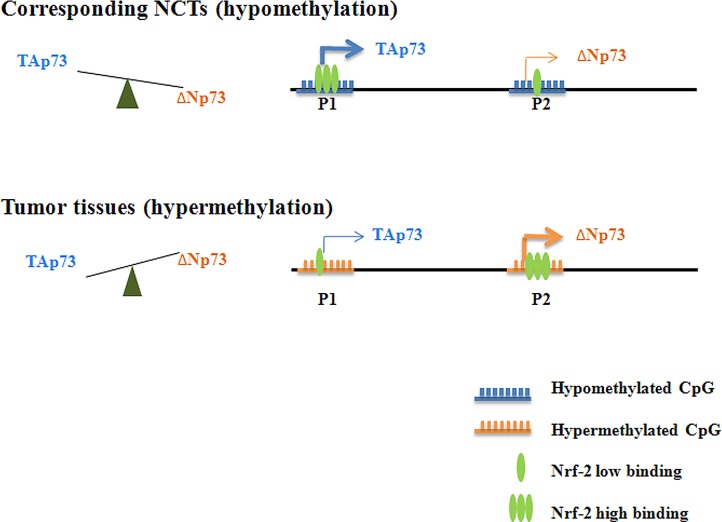
A schematic model of dynamic regulation of TAp73 and ΔNp73 expression A schematic model of dynamic regulation of TAp73 and ΔNp73 expression by Nrf-2-mediated transcriptional activation and CpG island methylation induced transcriptional suppression. In normal breast tissue cells, both P1 and P2 promoters are non- or hypo-methylated, and they are easily accessible by transcription factors such as Nrf-2. When Nrf-2 binds to its binding site in P1, transcription of TAp73 isoform will be activated. The same should apply to ΔNp73. However, since P1 is upstream of P2, the active TAp73 transcription processes will interfere with Nrf-2 binding in P2. As a result, although P2 contains three Nrf-2 binding sites, the transcription of ΔNp73 isoforms will be inhibited. This leads to high TAp73 expression and low ΔNp73 expression. When P1 and P2 promoters are hypermethylated, they are less accessible to Nrf-2. Since P1 contains three CpG islands while P2 only contains one CpG island, the methylation induced transcriptional suppression affects TAp73 expression to a larger extent. Therefore, relatively enriched Nrf-2 binding at P2 promoter will activate abundant ΔNp73 transcription, while limited binding of Nrf-2 at P1 promoter leads to restricted TAp73 transcription. As a result, under a promoter hypermethylation state, ΔNp73 will be highly expressed while TAp73 expression remains low. This will eventually contribute to cell transformation and tumorigenesis.

Collectively our results suggest a model of dynamic regulation of TAp73 and ΔNp73 expression by Nrf-2-mediated transcriptional activation and CpG island methylation induced transcriptional suppression (Figure 6). In normal breast tissue cells, both P1 and P2 promoters are non- or hypo-methylated, and they are easily accessible by transcription factors such as Nrf-2. When Nrf-2 binds to its binding site in P1, transcription of TAp73 isoform will be activated. The same should apply to ΔNp73. However, since P1 is upstream of P2, the active TAp73 transcription processes will interfere with Nrf-2 binding in P2. As a result, although P2 contains three Nrf-2 binding sites, the transcription of ΔNp73 isoforms will be inhibited. This leads to high TAp73 expression and low ΔNp73 expression. When P1 and P2 promoters are hypermethylated, they are less accessible to Nrf-2. Since P1 contains three CpG islands while P2 only contains one CpG island, the methylation induced transcriptional suppression affects TAp73 expression to a larger extent. Therefore, relatively enriched Nrf-2 binding at P2 promoter will activate abundant ΔNp73 transcription, while limited binding of Nrf-2 at P1 promoter leads to restricted TAp73 transcription. As a result, under a promoter hypermethylation state, ΔNp73 will be highly expressed while TAp73 expression remains low. This will eventually contribute to cell transformation and tumorigenesis.

In brief, as shown in Figure 6, Nrf-2 can specifically bind to the P1 and P2 promoter, and a decreased TAp73 and an increased ΔNp73 expression were detected in tumor tissues which is detected hypermethylation [39], which was opposite to the expression in corresponding NCTs which is found hypomethylation. However, 5-aza-dC treatment led to an increased binding with P1 and a decreased binding with P2 promoter, resulting in a down-regulated ΔΝp73 and an up-regulated TAp73 expression in breast cancer cells lines.

In summary, our present results show that 5-aza-dC, a cytosine analogue designed to inhibit DNA methyltransferases, abolishes breast cancer cells growth advantages via cell cycle and apoptosis induced by increasing TAp73 and decreasing ΔNp73 expression. It is noteworthy that our data demonstrated for the first time that TAp73 and ΔNp73 are regulated by Nrf-2 through a regulatory region in the different promoters of p73 gene, and treatment with 5-aza-dC can enhance Nrf-2 to bind to the P1 promoter and inhibit Nrf-2 to bind to the P2 promoter resulting in the regulation of p73 expression, which was confirmed *in vitro* and *vivo*. To the best of our knowledge, this is the first report revealing that the up-regulation of TAp73 and down-regulation of ΔNp73 induced by 5-aza-dC are correlated with Nrf-2 expression in breast cancer cells. These findings would certainly highlight the potential for induction of TAp73 and inhibition of ΔNp73 as a promising target for treatment breast cancer and identify 5-aza-dC as an important candidate agent for future therapy. However, further larger studies and mechanistic investigations of the regulation mechanism of methylation on the expression of TAp73 and ΔNp73 are needed to validate our finding.

## MATERIALS AND METHODS

### Cell cultures, mice and 5-aza-dC treatment

Breast cancer cell lines MCF-7, SK-BR-3 and MDA-MB-231 purchased from the American Type Culture Collection (Manassas, VA, USA) were cultured in RPMI 1640 medium (GIBCO, Gaithersburg, MD, USA) supplemented with 10% fetal bovine serum (FBS) and 1% penicillin/streptomycin at 37°C in a humidified atmosphere with 5% CO2. Nrf-2 −/− and Nrf-2 +/+ (Wild Type) mice in C57BL/6 background were obtained from Jackson laboratory. The mice were housed in a temperature-controlled room with a controlled 12-h light/dark cycle. The mice were given free access to diet and water during the course of experiments. 5-aza-dC (Sigma) was dissolved in dimethylsulfoxide (DMSO, Sigma) and kept at -20°C as a 4mg/ml stock solution. The cells which plated in wells of 96-well dishes or 6-well dishes were treated with designated concentrations of drugs 5-aza-dC for 48h. 4-6 week-mice were used tail-intravenous injection with 250μg/kg of 5-aza-dC which dissolved in 200μl of PBS each 24h for twice. In control experiments, equal amounts of DMSO or PBS were added.

### Tissue samples

A total of 128 breast tumor tissues were collected from Ganzhou Tumor Hospital (Jiangxi, China) between Jan. 2008 and Jan. 2013. All patients did not receive chemotherapy or radiotherapy prior to surgery. Breast cancer tissue and corresponding noncancerous tissues-array (NCTs) sections containing BR802b (40 cancer cases and 40 NCTs) and BR804a (40 cancer cases and 40 NCTs) were bought from US Biomax (US Biomax, Inc.). All patients involved in this study consented to participate in the study and publication of its results. The experiments were approved by the Ethic Committee of Jinling Hospital and were conducted in compliance with the Helsinki Declaration. Disease histology was determined in accordance to the criteria of the World Health Organization. Pathologic staging was performed in accordance to the current International Union against Cancer tumor-lymph node-metastasis classification.

### Cell survival (MTT) assay

A total of 8×10^3^cells per well were seeded into 96-well plates and were incubated with various concentrations of drugs 5-aza-dC for 48h. The number of viable cells was measured following addition 10μl of 0.5 mg/ml MTT solution (Sigma) in each well, and then the medium was replaced with 100μL DMSO after 4h and vortexed for 10min. Absorbance was measured at 490nm with a microplate reader (BIO-RAD, USA). Each assay was performed in triplicate.

### Cell cycle analysis

A cell cycle test Detection Kit (KeyGEN Biotech, CA) was used to detect cell cycle according to the manufacturer's instructions. MCF-7, SK-BR-3 and MDA-MB-231 were treated with 5-aza-dC from 0 to 40μmol/L for 48h, then harvested by trypsinization (not with EDTA), washed with PBS, and fixed with 500μL iced cold 70% ethanol at 4°C. After 4h, the cells were washed with PBS, dissolved in 100μL RNase, and incubated for 30min at 37°C, then added 400μL Propidium Iodide and analyzed with flow cytometer (BD FACS calibur, USA) within 1 h. Each sample was tested in triplicates and untreated cells were used as controls.

### Apoptosis analysis

An annexin V-fluorescein isothiocyanate (FITC) apoptosis detection kit (KeyGEN Biotech, CA) was used to detect apoptosis according to the manufacturer's instructions. Briefly, breast cancer cells from each treatment group were incubated in 6-well dishes for 48h, harvested include cells at supernatant, centrifuged and washed with PBS. Cell pellets were resuspended and incubated in 500μl binding buffer. After 15 minutes incubated with 5μ1 annexin V-fluorescein isothiocyanate (FITC) and 5μ1 propidium iodide (PI) in the dark at room temperature, the apoptotic cells (FITC+/PI −) were monitored with a flow cytometer (BD FACS calibur, USA). Each sample was tested in triplicate and untreated cells were used as controls.

### Measurement of DNMTs activity

EpiQuik™ Nuclear Extraction Kit I (Epigentek, Brooklyn, NY, USA) was used to isolated nuclear protein after breast cancer cells exposed to designated concentrations of 5-aza-dC. After protein quantification with BCA kit (Thermo Scientific), 5μg of nuclear protein was used to measure total DNMT activity with the EpiQuik™ DNA Methyltransferase Activity/Inhibition Assay (Epigentek) according to the manufacturer's instructions, and absorbance was measured at 450nm/499nm with microplate reader (BIO-RAD, USA), the formula of DNMTs activity is as follows: DNMT activity (OD/h/mg) =1000×(Sample OD – Blank OD) /Protein amount (μg)/h

### DNA extraction and methylation analysis

MCF-7 was incubated with 20μmol/L 5-aza-dC or DMSO for 48h in 6-well dishes, harvested and centrifuged. Genomic DNA was extracted from the Cell pellets using the DNeasy kit (Biotech, CA) according to the manufacturer's protocol. Quality and quantity of the DNA was assessed by spectrophotometry at 260/280 nm. 1μg of genomic DNA was treated with sodium bisulfate, using the EZ DNA methylation Kit (ZymoResearch, CA, USA). Pyrosequencing was performed as the report described [40]. The methylation index (MtI) for P1 promoters and P2 promoter were calculated as the average methylation% of the examined CpGs.

### RNA extraction, cDNA synthesis and mRNA expression analysis by RT-PCR

Total RNA was extracted from the cultured cells using Trizol (Invitrogen, CA, USA) according to the manufacturer's protocol, reverse transcribed using a PrimeScript 1st Strand cDNA synthesis kit (TaKaRa, Dalian, China) according to the manufacturer's instructions. Primers were designed for TAp73, ΔNp73 and GAPDH and were synthesized by Invitrogen (Carlsbad, CA) as follows: TAp73(5′-CCAGGCTCTCTTTCAGCTTCA-3′ and 5′-GACGGAATTCACCACCATCCT-3′), product size: 389bp; ΔNp73 (5′-GCCACGGCCCAGTTCAAT-3′ and 5′-GAAGGTGGAGCTGGGTTGTG-3′), product size: 138bp; GAPDH: 5′-CCATGGAGAAGGCTGGGG-3′ and 5′-CAAAGTTGTCATGGATGACC-3′, product size: 195bp. PCR analysis was performed in a 25uL volume with amplification conditions: 95°C for 2 min, [94°C for 30 s, 56°C for 45 s and 72°C for 45 s] 35 cycles, 72°C for 10 min. PCR products were separated on 1.5% agarose gels, stained with ethidium bromide and photoEach. GAPDH was used as loading control.

### Western blotting

Whole cell lysates were prepared from 5-aza-dC treated cells and untreated controls as previously described. Total protein was extracted using RIPA buffer supplemented with protease and phosphatase inhibitors and quantitied using BCA kit (Thermo Scientific). 20μg proteins which were loaded per lane were separated on a sodium dodecylsulfate-polyacrylamide gel and blotted onto nitrocellulose. Blots were blocked with 5% dry milk in tris-buffered saline/0.1% tween-20 and incubated overnight with a diluted solution of primary antibody at 4°C, and then with the horseradish peroxidase-conjugated secondary antibody (1:5000) for 2 h. The specific antibodies used for Western blot were mouse anti-p73 antibody (ab17230), mouse anti-p73 Delta N antibody (ab13649), rabbit anti-Nrf-2 antibody (ab62352); Bands were normalized to GAPDH or β-actin expression which was used as an internal loading control. Results from at least two separate experiments were analyzed.

### Immunofluorescence analysis

Immunofluorescent staining was used to verify the protein expression and examine the subcellular localization of TAp73 and ΔNp73. Cells were plated onto glass coverslips in 6-well plates and treated with 20μmol/L 5-aza-dC for 48h. The cells were washed with PBS and fixed in 4% paraformaldehyde for 20min, permeabilized with 0.1% TritonX-100 for 10min, cells were incubated 1h at 37°C with the following antibody: mouse anti-p73 antibody (1:100), mouse anti-p73 Delta N antibody (1:100). The cells were then washed with PBS and incubated for 30min at 37°C with Secondary antibodies anti-mouse IgG conjugated with FITC (Invitrogen; 1:200). Subsequently, nuclei were counterstained with 4′, 6-diamidino-2-phenylindole (DAPI) for 10min. Samples were photographed on a fluorescent microscope (Aiovert 200; Carl Zeiss).

### Plasmids and transient transfection

Plasmids pcDNA3-EGFP-C4-Nrf-2 was purchased from addgene (USA). The new plasmid was named as p-Nrf-2. The vector pGPU6/GFP/Neo used for cloning Nrf-2 short hairpin RNA (shRNA) was purchased from GenePharma (Shanghai, China). The target sequence was GGGAGGAGCTATTATCCATTC. The new plasmid was named as sh-Nrf-2. Random sequence was used as negative control. MCF-7 cells were plated in 6-well plates at a density of 1×10^6^ cells/plate 24 h prior to transfection. Then p-Nrf-2, sh-Nrf-2, and random sequence were transfected by TurboFect Transfection Reagent (Thermo Scientific) according to the manufacturer's protocol. After incubation at 37°C 24h, cells were collected to testify the expression of TAp73 and ΔNp73 by Western blot analysis.

### ChIP assay

The ChIP assay was performed using a kit from Beyotime (China). Briefly, 70% confluent MCF-7 cells were treated with DMSO or 20μmol/L 5-aza-dC for 24h and then fixed in 1% formaldehyde for 15 min. Cells were lysed, and nuclei were pelleted by centrifugation. Nuclei were resuspended and sonicated on ice using a sonicator to shear the cross-linked DNA to an average length of 200–1000 bp and centrifuged at 12,000 rpm to remove insoluble material. Sheared chromatin was immunoprecipitated with 1μg of anti-Nrf-2 (Santa Cruz Biotech, USA) or control IgG antibody overnight at 4°C. The cross-links were reversed with proteinase K in ChIP Elution buffer for 1h at 62°C. PCR amplification detected the forecasted regions of P2 promoter bound to Nrf-2 using 1μl of each of the purified DNA. Primers were designed for TAp73, ΔNp73-p-1 and ΔNp73-p-2 as follows: TAp73: (5′-AGAGCTTGAATACCTCGGAGAAGTT-3′ and 5′- TTTGAGGTAAGGTTCTCGGGTC-3′), product size: 279bp; ΔNp73-p-1 (5′-AGACGCCCTTCCTGAACCTGAT-3′ and 5′-CTGAGGACGAAAGGACGATT-3′), product size: 224bp; ΔNp73-p-2 (5′-AGGAAAGGGGAAAGGGTCTC-3′ and 5′-CATTGTATTTCAGCCGTCTTGG-3′), product size: 212bp. PCR analysis was performed in a 25uL volume with amplification conditions: 95°C for 5min, [95°C for 15s, 56°C for 30s and 72°C for 30s] 35cycles, 72°C for 10min. PCR products were separated on 2% agarose gels, stained with ethidium bromide and photoEach. GAPDH was used as loading control. The relative bindings of Nrf-2 to the region of P2 promoter were also measured by Q-PCR with custom exon junction spanning BRYT primers using a 7500 Real-Time PCR System (Applied Biosystems) according to the manufacturer's instructions of GoTaqR qPCR Master Mix (Promega, USA).

### Immunohistochemistry

128 breast tumor tissues and TMA sections were de-paraffinized with xylene. Heat-mediated antigen retrieval was fulfilled with citrate buffer (BioGenex Laboratories, San Ramon, CA). Antibody staining was visualized with DAB (Sigma, D-5637) and hematoxylin counterstain. The H-score method was used in this trial; we multiplied the percentage score by the staining intensity score. The percentage of positively stained cells was scored as “-” (0%), “+” (1%-25%), “++” (26%-50%), “+++” (51%-100%). Intensity was scored as “-”: negative, “+”: weak, “++”: moderate and “+++”: strong. Immunohistochemical scoring was performed without prior knowledge of the clinical response. 55 pair cases including invasive ductal carcinoma and corresponding cancer adjacent normal breast tissue were selected to further study after removing the unqualified cases in TMA sections. Immunostained sections were scanned using a microscope (Aiovert 200; Carl Zeiss).

### Statistical analysis

The SPSS Statistics 16.0 (SPSS Inc.) was used for statistical analysis. Data were analyzed using one-way ANOVA or a Student's t-test. Data are presented as means ± SD of three independent experiments. The statistical significance was defined by *P < 0.05 or **P<0.001, and are shown in the figures. For IHC data, Two-Related-samples test between breast cancer and NAT was used, and the statistical significance of the correlation between ΔNp73 expression level and Nrf-2 expression level in breast cancers or in NAT was estimated by using the Spearman's correlation analysis, *P < 0.05 or **P<0.001.

## SUPPLEMENTARY MATERIAL FIGURE AND TABLE


