# Supplementary material for: Transcriptional regulation of the p73 gene by Nrf-2 and promoter CpG methylation in human breast cancer
Source: Oncotarget. 2014 Jul 17;5(16):6909–22. doi: 10.18632/oncotarget.2230 (PMC4196172; doi:10.18632/oncotarget.2230)
Supplement: Supplementary file 1 [file oncotarget-05-6909-s001.pdf]

Transcriptional regulation of the p73 gene by Nrf-2 and promoter CpG methylation in human breast cancer

Supplementary Material

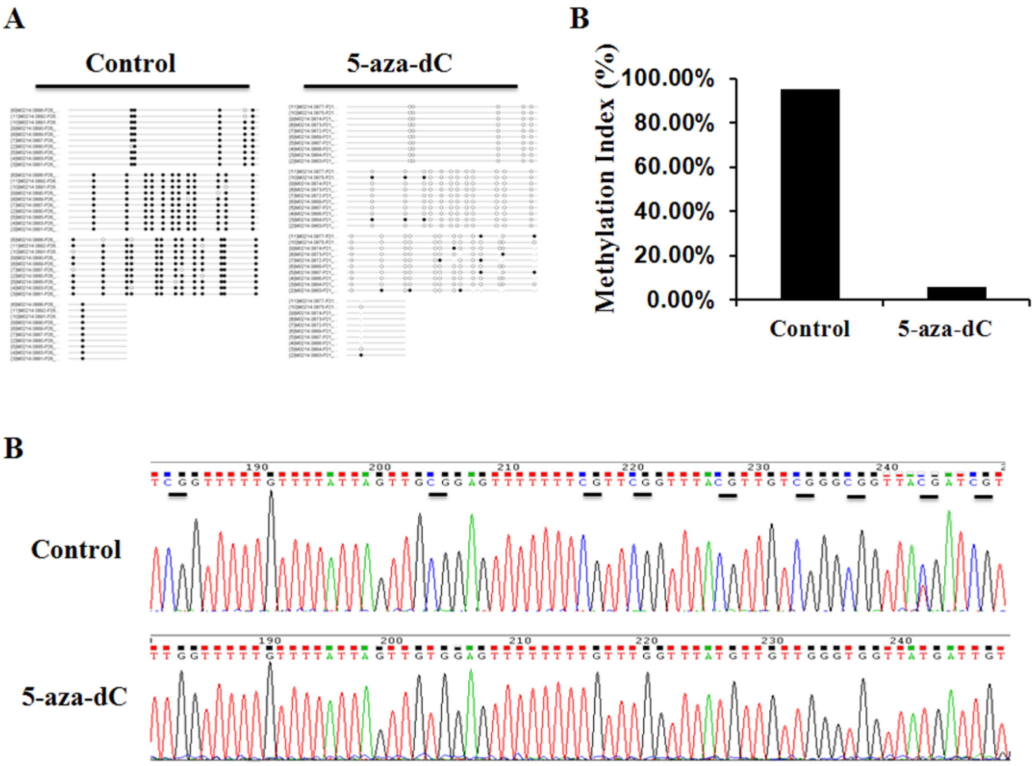

Figure S1: Bisulfite sequencing analysis of P2 promoter in MCF-7 cell line. (**A**) bisulfite sequencing analysis (BSP) was conducted on bisulfite-modified DNA from P2 in MCF-7 cultured with 20μmol/L 5-aza-dC or DMSO. Result of sequence analysis of ten independent clones; percent methylation across all 31 CpG dinucleotides within the 400-bp amplicon. ●, methylated CpG; ○, unmethylated CpG. (**B**) The relative methylation index (x%) for P2 are represented by column graph. (**C**) partial sequence of P2 using bisulfite sequencing analysis.

Table S1: TAp73, ΔNp73 and Nrf-2 expression in breast cancer and NCTs microarray. TAp73, ΔNp73 and Nrf-2 expression in breast cancer and NCTs microarray with clinicopathological parameter including age, grade, stage and TNM.

					Tumor				NCTs		
Age	Grade	Stage	TNM	Site	TAp73	DNp73	Nrf-2	Site	TAp73	DNp73	Nrf-2
48	1	IIa	T2N0M0	G7	1	0	0	G8	2	0	0
46	2	IIIa	T3N2M0	A8	0	0	0	E8	1	0	1
34	2	IIIb	T4N0M0	D1	1	0	0	D2	1	0	1
42	2	IIa	T1N1M0	H7	0	0	0	H8	0	0	0
50	2	IIb	T2N1M0	A6	0	0	0	E6	0	0	0
52	2	IIb	T2N1M0	F1	0	1	0	F2	0	0	1
39	2	IIb	T2N1M0	D1	0	2	0	H1	1	0	0
38	2	IIIb	T4N0M0	C9	0	2	0	G9	0	0	0
49	2	IIIb	T4N0M0	D9	1	2	0	D10	0	0	0
48	2	IIb	T3N0M0	D4	1	0	0	H4	1	1	0
38	2	IIb	T2N1M0	C9	1	0	0	C10	0	1	0
50	2	IIa	T2N0M0	B6	0	1	0	F6	1	1	1
41	1	IIb	T2N1M0	C5	1	2	0	C6	2	1	0
37	1	IIa	T2N0M0	E5	0	2	0	E6	1	1	1
47	2	IIb	T2N1M0	C1	0	2	0	C2	0	1	0
40	2	IIIa	T2N2M0	G9	0	3	0	G10	0	1	0
56	2	IIIa	T2N2M0	E9	1	0	1	E10	1	0	0
30	2	IIa	T2N0M0	C7	0	0	1	C8	0	0	0
40	3	IIa	T2N0M0	A1	0	0	1	E10	0	0	0
36	1	IIa	T2N0M0	E3	1	0	1	E4	0	0	2
35	2	IIa	T2N0M0	B9	0	1	1	B10	1	0	0
51	-	I	T1N0M0	A9	0	1	1	E9	1	0	0
39	1	IIa	T2N0M0	A1	0	1	1	E1	0	0	0
56	2	IIIa	T3N2M0	H1	0	2	1	H2	1	0	0
50	2	IIIa	T2N2M0	H5	0	2	1	H6	1	0	0
40	2	IIb	T2N1M0	A4	0	2	1	E4	1	0	0
38	1	IIa	T2N0M0	B7	0	2	1	F7	1	0	0
49	2	IIb	T3N0M0	A7	0	2	1	A8	0	0	1
51	2	IIIb	T4N0M0	F7	0	2	1	F8	0	0	0
39	2	IIIa	T3N1M0	H3	0	2	1	H4	0	0	1
50	2	IIb	T2N1M0	B8	0	2	1	F8	0	0	0
64	2	IIa	T2N0M0	D3	0	2	1	H3	0	0	0
52	3	IIa	T2N0M0	D5	0	3	1	D6	1	0	0
45	2	I	T1N0M0	B5	0	3	1	F5	0	0	0
44	2	IIb	T2N1M0	B1	0	3	1	F10	0	0	1
36	2	IIb	T2N1M0	B2	1	0	1	F2	0	1	0
45	1	IIb	T2N1M0	C1	1	2	1	G1	2	1	0
44	1	IIIa	T2N2M0	A2	0	2	1	E2	1	1	0
39	3	IIa	T2N0M0	B1	0	2	1	F1	1	1	0
48	2	IIa	T2N0M0	C7	0	2	1	G7	1	1	0
40	2	IIb	T2N1M0	C8	0	2	1	G8	1	1	1
48	2	IIa	T2N0M0	C1	0	2	1	G10	1	1	1
32	2	IIb	T3N0M0	G1	0	2	1	G2	0	1	1
48	2	IIIb	T4N0M0	B4	1	3	1	F4	0	2	1
40	2	I	T1N0M0	A3	0	0	2	E3	0	0	1
30	2	IIIa	T2N2M0	B1	0	2	2	B2	1	0	1
36	2	IIb	T2N1M0	E1	0	2	2	E2	0	0	0
60	2	IIIb	T4N2M0	C3	3	3	2	G3	0	0	1
44	2	IIb	T2N1M0	A7	1	3	2	E7	2	1	0
36	1	IIa	T2N0M0	F3	0	3	2	F4	1	1	1
43	2	IIa	T2N0M0	B3	0	3	2	F3	1	1	1
52	2	IIb	T2N1M0	B9	0	3	2	F9	1	1	1
39	2	IIb	T2N1M0	C5	0	3	3	G5	1	0	2
48	3	IIa	T2N0M0	C2	1	0	3	G2	2	1	2
52	-	IIa	T2N0M0	A5	0	2	3	E5	2	1	0
